# Retraction: Polysaccharide IV from *Lycium barbarum* L. improves lipid profiles of gestational diabetes mellitus of pregnancy by upregulating ABCA1 and downregulating sterol regulatory element-binding transcription 1 *via* miR-33

**DOI:** 10.3389/fendo.2022.1130062

**Published:** 2023-01-06

**Authors:** Frontiers Editorial Office

The journal retracts the 23 February 2018 article cited above.

Following publication, concerns were raised regarding the integrity of the images in the published figures. The authors failed to provide a satisfactory explanation during the investigation, which was conducted in accordance with Frontiers’ policies.

This retraction was approved by the Chief Editors of Frontiers in Endocrinology and the Chief Executive Editor of Frontiers. The authors agree to this retraction.

